# Combining germline, tissue and liquid biopsy analysis by comprehensive genomic profiling to improve the yield of actionable variants in a real-world cancer cohort

**DOI:** 10.1186/s12967-024-05227-2

**Published:** 2024-05-15

**Authors:** I. Vanni, L. Pastorino, V. Andreotti, D. Comandini, G. Fornarini, M. Grassi, A. Puccini, E. T. Tanda, A. Pastorino, V. Martelli, L. Mastracci, F. Grillo, F. Cabiddu, A. Guadagno, S. Coco, E. Allavena, F. Barbero, W. Bruno, B. Dalmasso, S. E. Bellomo, C. Marchiò, F. Spagnolo, S. Sciallero, E. Berrino, P. Ghiorzo

**Affiliations:** 1 Genetics of Rare Cancers, IRCCS Ospedale Policlinico San Martino, 16132 Genoa, Italy; 2 Department of Internal Medicine and Medical Specialties (DiMI), University of Genoa, 16132 Genoa, Italy; 3 Medical Oncology Unit 1, IRCCS Ospedale Policlinico San Martino, 16132 Genoa, Italy; 4 Medical Oncology Unit 2, IRCCS Ospedale Policlinico San Martino, 16132 Genoa, Italy; 5 Pathology Unit, Department of Surgical Sciences and Integrated Diagnostics (DISC), University of Genoa, 16132 Genoa, Italy; 6 Pathology Unit, IRCCS Ospedale Policlinico San Martino, 16132 Genoa, Italy; 7 Lung Cancer Unit, IRCCS Ospedale Policlinico San Martino, 16132 Genoa, Italy; 8 Pathology Unit, Candiolo Cancer Institute, FPO - IRCCS, 10060 Candiolo, Turin Italy; 9 Department of Medical Sciences, University of Torino, 10060 Turin, Italy; 10 Plastic Surgery, Department of Surgical Sciences and Integrated Diagnostics (DISC), University of Genoa, 16132 Genoa, Italy

**Keywords:** Next-generation sequencing, Circulating cell-free DNA, Germline pathogenic variants, Droplet digital pcr (ddPCR), Targeted therapy, Clinically actionable variants

## Abstract

**Background:**

Comprehensive next-generation sequencing is widely used for precision oncology and precision prevention approaches. We aimed to determine the yield of actionable gene variants, the capacity to uncover hereditary predisposition and liquid biopsy appropriateness instead of, or in addition to, tumor tissue analysis, in a real-world cohort of cancer patients, who may benefit the most from comprehensive genomic profiling.

**Methods:**

Seventy-eight matched germline/tumor tissue/liquid biopsy DNA and RNA samples were profiled using the Hereditary Cancer Panel (germline) and the TruSight Oncology 500 panel (tumor tissue/cfDNA) from 23 patients consecutively enrolled at our center according to at least one of the following criteria: no available therapeutic options; long responding patients potentially fit for other therapies; rare tumor; suspected hereditary cancer; primary cancer with high metastatic potential; tumor of unknown primary origin. Variants were annotated for OncoKB and AMP/ASCO/CAP classification.

**Results:**

The overall yield of actionable somatic and germline variants was 57% (13/23 patients), and 43.5%, excluding variants previously identified by somatic or germline routine testing. The accuracy of tumor/cfDNA germline-focused analysis was demonstrated by overlapping results of germline testing. Five germline variants in *BRCA1*, *VHL*, *CHEK1, ATM* genes would have been missed without extended genomic profiling. A previously undetected *BRAF* p.V600E mutation was emblematic of the clinical utility of this approach in a patient with a liver undifferentiated embryonal sarcoma responsive to BRAF/MEK inhibition.

**Conclusions:**

Our study confirms the clinical relevance of performing extended parallel tumor DNA and cfDNA testing to broaden therapeutic options, to longitudinally monitor cfDNA during patient treatment, and to uncover possible hereditary predisposition following tumor sequencing in patient care.

**Supplementary Information:**

The online version contains supplementary material available at 10.1186/s12967-024-05227-2.

## Background

Next-Generation Sequencing (NGS) applications are a powerful tool to aid clinical decision-making in the management of cancer patients, opening new opportunities for precision oncology and precision prevention approaches [1]. NGS approaches include the analysis of panels of various sizes, ranging from a few tens up to hundreds of genes. These latter Comprehensive Panels (CPs) are increasingly being implemented in molecular diagnostics to identify targets of innovative therapeutic strategies for patients who may benefit the most from this approach [2]. However, the actual detection yield in terms of actionable variants is still to be clarified, with evidence suggesting that rare cancers may be enriched for actionable variants [3, 4]. In this context, NGS application in clinical practice requires the implementation of multidisciplinary Molecular Tumour Boards (MTBs) [5, 6]. The MTBs’ goal is to evaluate other potential therapeutic approaches, upon failure or absence of available therapy lines, based on the identified molecular alterations and the clinical significance of the available drugs. The NGS CPs suitable for this purpose cover all genetic-molecular alterations relatable to a clinical indication, hence Single Nucleotide Variants (SNVs), Insertions/Deletions (InDels), Copy Number Variations (CNVs) and structural rearrangements. NGS CPs may also reveal germline variants associated with cancer susceptibility syndromes, for which tailored surveillance protocols can be proposed.

Comprehensive NGS analysis should be performed prioritizing patients with advanced-stage tumors, preferably on tumor tissue. However, circulating cell-free DNA (cfDNA) could represent an alternative when the tissue is unavailable, inadequate, or should comorbidities be hindering invasive tissue collection. cfDNA analysis, commonly known as liquid biopsy, has been introduced into clinical practice for non-invasive genome analysis, treatment response monitoring, identification of drug-resistant mechanisms, early detection of recurrence, and intra-tumoral heterogeneity, compensating for the limitations of the NGS performed on tumor tissues.

At the present time, the only FDA-approved cfDNA NGS CPs are: Guardant360® CDx (Guardant Health, Inc.; Redwood, CA, USA) (G360)] and FoundationOne® LiquidCDx (FoundationMedicine, Inc.; Cambridge, MA, USA) (F1LCDx) [7–9]. Nevertheless, other NGS CPs, able to identify SNVs, InDels, gene rearrangements, CNVs, Tumor Mutational Burden (TMB) scoring and microsatellite status, could be implemented in the clinical research setting, such as the TruSight Oncology (TSO) 500 ctDNA assay.

Our aim was to analyse, through NGS CPs, a consecutive series of matched cancer tissues/blood samples belonging to patients eligible for germline and somatic molecular profiling, in order to identify targets for novel or non-standard therapeutic strategies and determine the actual detection yield of actionable variants. In addition, we also evaluated the feasibility and sensitivity of extended genomic profiling by liquid biopsy and compared the results with those obtained from tumor tissue analysis.

Here we show how this integrated approach can benefit both patients in need of additional therapeutic options, and patients with rare and possibly hereditary tumors, for whom individual risks could be assessed for tailored surveillance.

## Methods

### Biological samples

All patients’ samples were consecutively collected, under local IRB-approved protocols (CER 595/2020), from patients, mainly with metastatic tumors, treated or in follow-up at our center (IRCCS Ospedale Policlinico San Martino in Genoa, Italy) over a twelve-month period. Inclusion criteria were patients with: (1) absence/exhaustion of approved treatment lines or resistant disease; (2) long responding tumor who were deemed fit for other potential treatments; (3) rare tumor; (4) suspected hereditary cancer; (5) primary tumor with high metastatic potential; (6) metastatic tumor of unknown primary origin. Somatic DNA/RNA were extracted from fresh tissue and/or Formalin-Fixed Paraffin-Embedded (FFPE) sections for each patient included in the study, when possible. FFPE tumor samples were selected and revised by the pathology team of our institute based on tissue quality and tumor cellularity. A peripheral blood sample was also collected from all patients for germline DNA and cfDNA extraction.

### DNA/RNA and circulating cell-free DNA (cfDNA) extraction

Genomic DNA (gDNA) was extracted from peripheral blood using the Diatech MagCore® HF16Plus (RBC Bioscience, New Taipei City, Taiwan) with the Genomic DNA Large Volume Whole Blood kit. Somatic DNA and RNA from FFPE tissue were extracted from the tumor sections using the AllPrep DNA/RNA FFPE Kit (QIAGEN, Valencia, CA, USA) according to the manufacturer’s instructions. Somatic DNA from fresh tissue biopsies was isolated using DNeasy® Blood & Tissue Kit (QIAGEN, Valencia, CA, USA) while the somatic RNA was extracted by the Tissue Lyser plus Maxwell® RSC simplyRNA Tissue Kit (AS1340 Promega, Southampton, UK) in accordance with the manufacturer. Quantity and purity of the tumor and genomic DNA were examined by SPECTROstar Nano (BMG Labtech, Offenburg, Germany) to measure the whole absorption spectrum (220–750 nm) and calculate absorbance ratios at both 260/280 and 260/230. Moreover, all somatic and germline samples were also quantified by Qubit® 2.0 Fluorometer (Invitrogen, Carlsbad, CA, USA) and Agilent 2200 TapeStation system using the Genomic DNA ScreenTape assay (Agilent Technologies, Santa Clara, CA, USA). The Infinium FFPE DNA QC Kit (Illumina) evaluated the somatic DNA quality, and only samples that passed the established threshold were used in the subsequent experiments.

Total RNA concentration was quantified by the Qubit® 2.0 Fluorometer (Invitrogen) using Qubit™ RNA High-Sensitivity (HS) Assay Kits (ThermoFisher, Waltham, MA, USA), while FFPE RNA quality was assessed by the and Agilent 2200 TapeStation system using the Agilent High Sensitivity RNA ScreenTape (Agilent Technologies, Santa Clara, CA, USA) to calculate the DV200% (percentage of RNA fragments > 200 nucleotides).

cfDNA was isolated from 5 to 14 mL of plasma using MagMAX™ Cell-Free DNA Isolation Kit according to the manufacturer’s instructions (ThermoFisher Scientific) and quantified using the Qubit® dsDNA HS Assay Kit on the Qubit 2.0 fluorometer (ThermoFisher Scientific). The purity and quantity of DNA size fragments were analyzed by the Agilent High Sensitivity DNA Analysis Kit (Agilent Technologies) using TapeStation 2200 instrument (Agilent Technologies).

### Next-generation sequencing (NGS) targeted sequencing

Somatic DNA/RNA samples were subjected to deep sequencing using the TruSight Oncology 500 (TSO500) panel (Illumina, San Diego, California, U.S.) starting from a quantity input between 44 and 285 ng. The targeted panel is 1.94 Mb in size, encompassing the exon sequence of mostly cancer-related 523 genes (coding size 1.2 Mb) (Additional file 1). The panel allows the assessment of MicroSatellite Instability (MSI) status (approximately 120 loci), TMB, and CNVs data (about 59 genes), following the manufacturer’s protocol. Libraries were sequenced on a NextSeq 500 instrument (Illumina, San Diego, California, USA) to reach a minimum of 500X read depth. Raw data were processed by the Illumina Local App associated with the TSO500 panel (TruSight Oncology 500 *v*2.2 Local App) to produce.fastq files through the alignment of the sequence to the human reference sequence GRCh37 (hg19). The Local App also performed sequencing QCs and somatic variant calling with a tumor-only pipeline. The TMB was assessed as the number of eligible variants according to the specific Illumina Local App parameters divided by the panel size. TMB eligible variants are calculated as follows: (*i*) Variants in the coding region (RefSeq Cds), (*ii*) Variant Allele Frequency (VAF) ≥ 5%, *iii*) coverage ≥ 50X, (*iv*) SNVs and short InDels (Multi-Nucleotide Variants (MNVs) excluded)), (*v*) nonsynonymous and synonymous variants, (*vi*) variants with COSMIC count ≥ 50 excluded. The MSI status is reported as a sample-level microsatellite score compared to an internal set of normal samples. MSI is called if the mean distance difference is greater than or equal to the default threshold (0.1) and the p-value is less than or equal to the default threshold (0.01).

gDNA were analysed with the TruSight Hereditary Cancer Panel (Illumina) including 10,341 probes that target 113 cancer predisposition genes (Additional file 1). The panel is 403 kb in size and covers all exons of 114 genes and 125 Single-Nucleotide Polymorphisms (SNPs). Libraries were sequenced on a NextSeq 500 instrument (Illumina, San Diego, California, USA) to reach a minimum of 100X read depth. fastq files and the resulting data were analyzed using the Dragen platform on Basespace sequencing hub. Variant call format files were submitted to the Emedgene online platform and annotated using various public resources, as well as proprietary institutional databases and Emedgene’s proprietary resources.

cfDNA was sequenced using the TSO500 ctDNA Kit (Illumina). The panel provides the same analysis as the TruSight Oncology 500 (TSO500) (Additional file 1). Libraries were constructed using a minimum 30 ng of cfDNA. Indexed pre-capture libraries were enriched for specific targeted regions covered by the TruSight Oncology 500 ctDNA kit by two rounds of hybridization, streptavidin bead capture and clean up. The enriched libraries were amplified, purified with sample purification beads, and normalized with normalization beads prior to sequencing. Samples were sequenced on a NovaSeq 6000 instrument using Flowcell S4 2 × 150 cicli, XP protocol (1 pool per lane). cfDNA data analyses were performed using the DRAGEN TruSight Oncology 500 analysis software *v*2.1 with Illumina DRAGEN server *v*4. Moreover, fastq files and the resulting data were loaded on cloud via Illumina Connected Insight (ICI) for the secondary analysis.

### Somatic and germline variant calling, classification and filtering

For both bulk tumor and cfDNA analysis, the TruSight Oncology 500 analysis software *v*2.1 on the DRAGEN on-site server (*v*4.0, Illumina) applied a “tumor-only “pipeline.

Considering SNVs and small InDels, the Nirvana tool (https://github.com/Illumina/Nirvana) developed for variant calling and annotation is implemented in the TSO500 software. This software assesses the distribution of each variant within 3 germline databases (gnomAD exome *v*2.1, gnomAD genome v2.1, and 1000 Genomes Phase 3 *v*5a databases) and the somatic dataset COSMIC (v84). In particular, the “Germline” flag is added when a variant has been reported > 50 times in at least 2 germline databases, also taking into account the zygosity of the variant (VAF around 50% or 100%) and its distribution in the COSMIC somatic database. This information was summarized in the GermlineFilterDatabase annotation (TRUE: germline, FALSE: non-germinal). For all the variants, the pipeline added a second level of germline flagging, collapsed in the GermlineFilterProxi feature. This annotation is based on the statistical evaluation of the expected germline allele frequency of each variant, using the surrounding germline variants, assessing if the VAF of each variant is similar to the expected germline allele frequency. All variants annotated as TRUE (*i.e.* with a high probability for a germline status) were manually revised, and evaluated for the distribution within both germline and somatic databases provided by the DRAGEN server and an internal dataset of variants previously sequenced with the same methods (n = 145) [10–13].

All variants, both somatic and germline, were annotated with the Intervar tool [14], reporting the in-silico damaging evaluation (12 predictors related to the ANNOVAR tool) and the clinical significance reported in ClinVar (version 20221231 hg19 build). Actionability level of all the SNVs and small InDels were classified according to OncoKB (https://github.com/oncokb/oncokb-annotator) [14–16] and AMP/ASCO/CAP Tier classifications [17] by Illumina Connect Insight (ICI) Software. In particular, the druggable variants were selected based on the highest level of drug response evidence in OncoKB. To improve readability, we reported only OncoKB classification to describe each variant throughout the manuscript.

TSO500 and TSO500 ctDNA assays also allow the evaluation of CNV data for 59 genes. The pipeline normalized the counts on the target region with a panel of normal samples. GC bias correction applied a statistical model to calculate the scores of a CNV and predict copy number calls and Fold-Change (FC). These CNVs were annotated for the actionability level according to OncoKB input format, which follows the GISTIC 2.0 format: FC < 0.2 = − 2 (homozygous deletion); 0.2 < FC < 0.7 = − 1 (hemizygous deletion); 0.7 < FC < 1.5 = 0 (neutral/no change); 1.5 < FC < 2 = 1 (gain); FC > 2 = 2 (high level amplification) [18]. For the circulating tumor DNA (ctDNA) data, in which non-tumoral cfDNA could affect the Copy-Number (CN) calculation, we considered FC > 1.5 CN as high-level amplification (GISTIC value = 2). OncoPrint for data visualization were produced using the OncoPrint function of the ComplexHeatmap R package [19].

### Identification of the most confident variant allele frequency cut-off

To identify the lowest VAF cut-off for both bulk tumor and ctDNA analysis returning confident variants, we exploited the peculiarity of our cohort. First, we identified patients (n = 9) with TSO500 tissue sequencing and paired, sequenced ctDNA derived from a plasma sample temporarily close to surgery. For this sub-cohort (called In Time), we calculated the Jaccard index (JI) [20], a measure of concordance between the variants identified between two paired sequencing, for each couple of cut-offs (10% or 5% VAF for solid tumor DNA *vs* 10%/5%/1%/0.5%/0.1% for ctDNA). We obtained 9 JIs for each patient, and we compared the distribution of the concordance value for each intersection of cut-offs using the paired t-test. The lower intersection with a high level of confidence was applied to the rest of the cohort. The JI value was also used to define the concordance between the two sequencing approaches.

### Biological sample selected for the somatic clinical actionability detection yield

For patients whose cfDNA sample analysis was matched with tumor sampling within 4 months from plasma collection, the sum of actionable variants (AMP/ASCO/CAP Tier IA to IIC/ OncoKB level 1 to 3B) found both in cfDNA and tumor tissue was considered for the detection yield. On the contrary, only actionable variants found in the cfDNA analysis were included when the tumor tissue was collected more than 4 months before to plasma collection.

### Droplet digital PCR (ddPCR) analysis on circulating cell-free DNA (cfDNA)

*BRAF* V600 status was also evaluated by droplet digital PCR (ddPCR) for one patient (GE01) on cfDNA at five consecutive points during treatment. The presence of the *BRAF* V600 mutation and its allele frequency in the ctDNA was evaluated by the QX200 droplet digital PCR™ (ddPCR) system (Bio-Rad Laboratories, Inc., Hercules, CA, USA) using the “ddPCR BRAF V600 Screening Kit” (BioRad), able to detect p.Val600Glu, p.Val600Lys, and p.Val600Arg mutations in a single run.

## Results

### Study cohort

A total of 78 matched germline/tumor tissue/liquid biopsy DNA and RNA samples from 23 patients consecutively enrolled at our center were genomic profiled. All patients were of Caucasian ethnicity and the majority were female (15 of 23, 62%). Median age was 46 (range 32–85) years. Patients’ clinical and demographic characteristics, inclusion criteria and samples type analyzed in the study are presented in Table 1. Overall, eight rare cancers were present among the neoplasms diagnosed in our study cohort, namely undifferentiated embryonal sarcoma of the liver, lung atypical carcinoid tumor, breast neuroendocrine carcinoma, desmoplastic small round cell tumor, thymic carcinoma, leiomyosarcoma, cholangiocarcinoma, myxofibrosarcoma, but also others cancers such as melanoma and pancreatic cancer. Most patients had unavailable/exhausted lines of therapy or resistant disease (11 out of 23 patients). Three patients, 1 with urothelial carcinoma, 1 with renal carcinoma as part of Von-Hippel Lindau (VHL) syndrome and 1 with pancreatic ductal adenocarcinoma, were suspected to have a hereditary cancer syndrome. Two additional patients had gastro-intestinal metastatic cancer of unknown primary origin.

**Table 1:** Enrolled patients’ clinical and demographic characteristics, inclusion criteria and biological sample type analyzed in the study

Patient ID	Age (years)	Sex	Tumor Type	Stage at recruitment	Site of primary	Site of metastasis	Comprehensive genomic profiling	Study inclusion criteria
GE01^●^	37	F	Undifferentiated embryonal sarcoma of the liver	IV	Liver	Lung	G, T, LB	3
GE02	38	M	Pancreatic ductal adenocarcinoma	IV	Pancreas	Liver	G, LB	4
GE03	41	F	GI cancer	IV	Unknown (likely gastric or bilio-pancreatic)	Bone, peritoneum	G, T, LB	1, 6
GE04	62	M	Lung atypical carcinoid	IV	Lung	Bone, liver	G, LB	1, 3
GE05	46	F	Breast neuroendocrine carcinoma	III	Breast	–	G, LB	3
GE06^●^	85	F	Melanoma	IV	Scalp	Retroauricolar/periauricolar, liver	G, T, LB	1
GE07	32	M	Desmoplastic small round cell tumor	IV	Unknown	Liver, pleura, peritoneum, LN	G, T, LB	1, 3
GE08^●^	30	F	Renal carcinoma	IV	Kidney	Bone	G, T, LB	1
GE09	29	M	Thymic carcinoma	IV	Thymo	LN, pleura	G, T, LB	1, 3
GE10^●^	48	F	Leiomyosarcoma	IV	Inferior vena cava	Lung, chest	G, T, LB	3, 5
GE11	41	F	Cholangiocarcinoma	IV	Unknown (likely biliary origin)	Liver, LN, peritoneum	G, LB	1, 3
GE12^●^	40	F	Renal carcinoma (VHL syndrome)	IV	Pancreas, kidney (bilateral)	Lung	G, T, LB	2, 4
GE13	49	M	Rectal adenocarcinoma	loco-regional recurrence	Rectum	Pre-sacral	G,LB	1
GE14^●^	58	F	Melanoma	IV	Leg	Skin, brain	G, T, LB	1
GE15	50	M	Melanoma	IV	Leg	Brain, Skin, Small intestine	G, T, LB	2
GE16	79	F	Melanoma	IV	Leg	Skin	G, T, LB	1
GE17	61	F	Melanoma	IV	Trunk	Lung, breast, LN, mediastinum	G, LB	1
GE18	37	M	Urothelial carcinoma	IV	Bladder	LN, liver	G, LB	4
GE19^●^	40	M	GI cancer	IV	Unknown (likely upper GI tract)	Pleuric, mediastinum	G, T, LB	6
GE21	62	F	Pancreatic ductal adenocarcinoma	III	Pancreas	–	G, T, LB	2, 5
GE22^●^	70	F	Pancreatic ductal adenocarcinoma	III	Pancreas	–	G, T, LB	2, 5
GE23	34	F	Colon adenocarcinoma	IV	Right colon	Peritoneum, recto-vaginal septum	G, T, LB	5
GE24^●^	52	F	Myxofibrosarcoma	IV	Ileo-psoas	Peritoneum, abdominal wall, para-vescical adipose tissue	G, T, LB	3, 5

Enrolled patients and their biological samples analyzed in the study are shown. Patients ID with tumor tissue collected within 4 months from plasma collection are indicated with a black circle. All other tumor tissues are collected more than 4 months prior to plasma collection. The study inclusion criteria are: 1, absence/exhaustion of approved treatment lines or resistant disease; 2, long responding tumor who were deemed fit for other therapies; 3, rare tumor; 4, suspected hereditary cancer; 5, primary tumor with high metastatic potential; 6, metastatic tumor of unknown primary origin

*F* female, *M* Male, *VHL* Von Hippel-Lindau, *GI* gastro-Intestinal, *LN* lymph node, *G* germline, *T* tumor tissue, *LB* liquid biopsy

Germline DNA and cfDNA analysis were performed for all patients recruited in the study, while somatic DNA and RNA analysis on tumor tissue were carried out for 16 and 15 patients, respectively. Patients’ inclusion in the study coincided with the date of blood sampling for cfDNA analysis. Nine out of 16 tumor tissues were obtained within 4 months from plasma collection, while the remaining 7 were all obtained over 4 months prior to plasma collection (Table 1).

### Identification of the variant allele frequency (VAF) cut-off

The targeted panels applied for NGS sequencing (TSO500 for tissue and for ctDNA) potentially allowed the identification of SNVs and small InDels at very low frequency [21, 22].

To reduce as much as possible the inclusion of sequencing artefacts caused by formalin fixation, we adopted an adjusted VAF threshold exploiting the peculiarity of the cohort, that is paired tissue and cfDNA sequencing available for 9 patients. By intersecting all the variants called at different VAFs (10% or 5% for tissue and 10%, 5%. 1%, 0.5%, 0.1% for cfDNA), we calculated a correlation index, the Jaccard Index (JI), for each patient, and then compared the distribution of the JIs for each VAF combination. As represented in Additional file 2a and reported in Additional file 3, paired tests revealed the non-inferiority of the correlation index for the 5%_solid tumor_ and 0.5%_cfDNA_ (median JI = 0.91 ± 0.05), compared to the higher cut-off combinations, suggesting a high confidence in variant calling and a high level of concordance in variant calling between solid tumor and cfDNA sequencing approaches. The JI correlation showed a significant drop when we intersected the variants called at 0.1% of the cfDNA, regardless to the tissue VAF. However, as shown in Additional file 2b, we also observed that both this drop and the reduction in concordance between the sequencing approaches are sample-dependent, with at least 3/9 patients (GE10, GE01, and GE12) in which the JI increased by combining 0.1% _cfDNA_ and 5% _solid tumor,_ and with GE6 patient characterized by a cut-off independent lower correlation between cfDNA and solid tumor variant calling (JI = 0–82). Considering these data, we opted to include all variants with a 5% of VAF for the “TSO500 solid tumor”, and the 0.1% and 0.5% for the cfDNA SNVs/MNVs and InDels, respectively. Indeed, a manual revision for the cfDNA InDels variants comprised between 0.1 ≤ VAF < 0.5 with OncoKB levels 1 to 3 showed how these variants occurred within repeat base sequences and in more than one sample. Therefore, we considered those variants as sequencing artefacts.

### Tissue molecular profiling by the TruSight™ oncology 500 panel

**Fig. 1:**
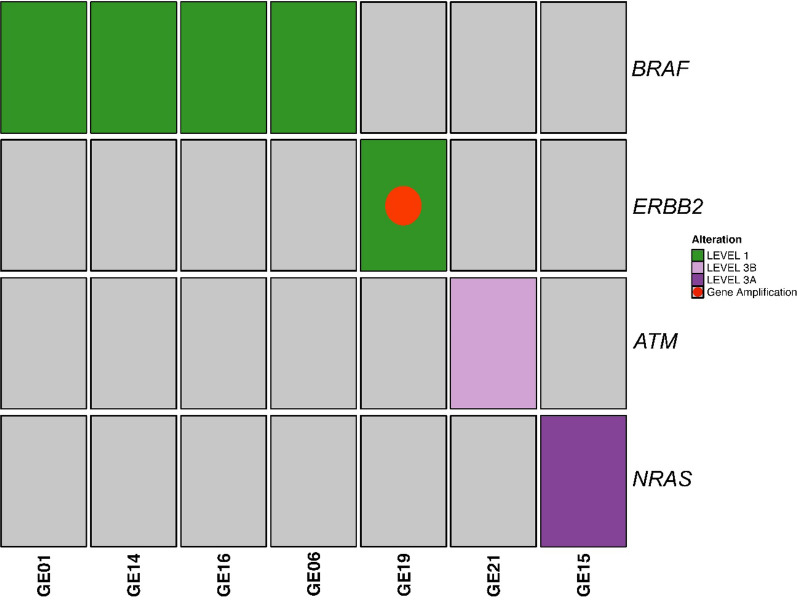
Actionable gene variants in 7 out of 16 tumor tissues analyzed. Actionable somatic variants and gene amplifications (OncoKB level 1–3) in 7/16 (43.75%) tumor tissues are reported. OncoKB level 1, 3B, 3A is indicated in green, lilac, purple, respectively. Gene amplification with OncoKB level 1 is represented with a red circle

We analyzed 16 tumor DNA and 15 tumor RNA from 16 out of 23 patients. No DNA tumor samples showed microsatellite instability (median MSI of 1.7), with TMB ranging from 0 to 32 mut/Mb (median TMB of 4.3) (Additional file 4). Among the somatic alterations (coding/nonsynonymous) detected with a VAF ≥ 5% in 13/16 tumors (Additional file 5), 11 mutations from 9 patients’ tumors were classified as potentially actionable according to OncoKB evidence levels (from 1, FDA-standard of care, to 4, hypothetical compelling biological evidence). Six out 11 mutations were classified as OncoKB level 1 to 3 (54.5%) of which 4 of them level 1 (33%) (Fig. 1, Table 2 and Additional file 6). CNV analysis revealed 4 potential amplifications (FC ≥ 2) (Additional files 7 and 8), but only one classified as OncoKB level 1 (*ERBB2* amplification in GE19 sample) (Fig. 1**, **Additional file 9). Overall, a total of 6 somatic mutations and 1 CNV were classified as OncoKB levels 1–3 in 7/16 patients (43.75%) (Fig. 1 and Table 2). Three of them had not been detected by previous routine testing (*ATM* p.R1730* in GE21 pancreatic tumor, *NRAS* p.Q61K in GE15 melanoma, and *BRAF* p.V600E in GE01 undifferentiated embryonal sarcoma of the liver).

**Table 2:** Results of integrated circulating cell-free DNA (cfDNA)/tumor tissue and germline analysis

Patient ID	cfDNA					Tissue					Germline	
	Gene	RefSeq	HGVS	VAF %/fold-change	OncoKB-AMP/ASCO/CAP classification	Gene	RefSeq	HGVS	VAF %/fold-change	OncoKB-AMP/ASCO/CAP classification	Gene	HGVS
GE01●	–	–	n.a	n.a	n.a	*BRAF*	NM_004333.4	p.V600E	51.4	1-IA	–	n.a
GE02	*FANCA*	NM_000135	p.W911Dfs*31	0.66	3B-IIC	n.d	n.a	n.a	n.a	n.a	–	n.a
GE05	*BRCA1°*	NM_007294.3	p.C64R	45.0	3B-IIC	n.d	n.a	n.a	n.a	n.a	*BRCA1*	p.C64R
GE06●	*BRAF*	NM_004333.4	p.V600R	0.13	1-IA	*BRAF*	NM_004333.4	p.V600R	24.8	1-IA	–	n.a
GE10●	*BRAF*	NM_004333.4	p.V600K	0.11	3B-IIC	–	n.a	n.a	n.a	n.a	–	n.a
GE11	*IDH2*	NM_002168.4	p.R172S	0.83	3B-IIC	n.d	n.a	n.a	n.a	n.a	–	n.a
	*NRAS*	NM_002524.4	p.Q61K	0.38	3B-IIC	n.d	n.a	n.a	n.a	n.a	–	n.a
	*MSH3°*	NM_002439	p.Q29X	47.3	n.a	n.d	n.a	n.a	n.a	n.a	*MSH3*	p.Q29X
GE12●	*VHL****°***	NM_000551.3	p.N131T	51.7	3B-IIC	VHL*	NM_000551.3	p.N131T	62.3	3B-IIC	*VHL*	p.N131T
GE14●	*IDH2*	NM_002168.4	p.R140Q	0.26	3B-IIC	–	n.a	n.a	n.a	n.a	–	n.a
	–	n.a	n.a	n.a	n.a	*BRAF*	NM_004333.4	p.V600E	28.7	1-IA	–	n.a
	*CHEK1°*	NM_001330427	p.E336Nfs*36	47.5	3B-IIC	CHEK1*	NM_001330427	p.E336Nfs*36	46.8	3B-IIC	*CHEK1*	p.E336Nfs*36
GE15	*NRAS*	NM_002524.4	p.Q61K	0.19	3A-IB	*NRAS*	NM_002524.4	p.Q61K	70.0	3A-IB	–	n.a
	*ATM°*	NM_000051.4	p.S1993Rfs*23	31.5	3B-IIC	ATM*	NM_000051.4	p.S1993Rfs*23	77.1	3B-IIC	*ATM*	p.S1993Rfs*23
GE16	–	n.a	n.a	n.a	n.a	*BRAF*	NM_004333.4	p.V600E	46.6	1-IA	–	n.a
GE17	*NRAS*	NM_002524.4	p.Q61K	38.9	3A-IIB	n.d	n.a	n.a	n.a	n.a	–	n.a
GE19●	*ERBB2*	NM_004448	Amplification	1.5	1-IA	*ERBB2*	NM_004448	amplification	7.2	1-IA	–	n.a
	*MUTYH°*	NM_001048174	p.G368D	48.6	n.a	MUTYH*	NM_001048174	p.G368D	49.6	n.a	MUTYH*	p.G368D
GE21	–	n.a	n.a	n.a	n.a	*ATM*	NM_000051.4	p.R1730*	5.4	3B-IIC	–	n.a
	*ATM°*	NM_000051.4	p.A1299Cfs*3	50.0	3B-IIC	ATM*	NM_000051.4	p.A1299Cfs*3	44.2	3B-IIC	*ATM*	p.A1299Cfs*3
GE22●	*NRAS*	NM_002524.4	p.G13D	0.43	3B-IIC	–	n.a	n.a	n.a	n.a	–	n.a

Somatic variants classified according to OncoKB and AMP/ASCO/CAP classification (1–3 or IA-IIC levels) and germline pathogenic/likely pathogenetic variants are shown*.* Patients ID with tumor tissue collected within 4 months from plasma collection are indicated with a black circle

*cfDNA* circulating cell-free DNA, *HGVS* Human Genome Variation Society nomenclature, *n.a.* not applicable, *n.d* not done, *–* wild-type

°This variant was classified as germline

RNA analysis, available for 15 patients, reported the AMP/ASCO/CAP Tier 1A (diagnostic) EWSR1-WT1 fusion in GE07 sample, confirming the diagnosis according to the pathology report (Ewing Sarcoma). The DSCRT-ESWR1 fusion was also validated with the FusionPlex Expanded Sarcoma—v1.1 (Archer, Boulder, CO, USA) NGS panel.

### Circulating cell-free DNA molecular profiling by TruSight™ Oncology (TSO) 500 ctDNA gene panel

**Fig. 2:**
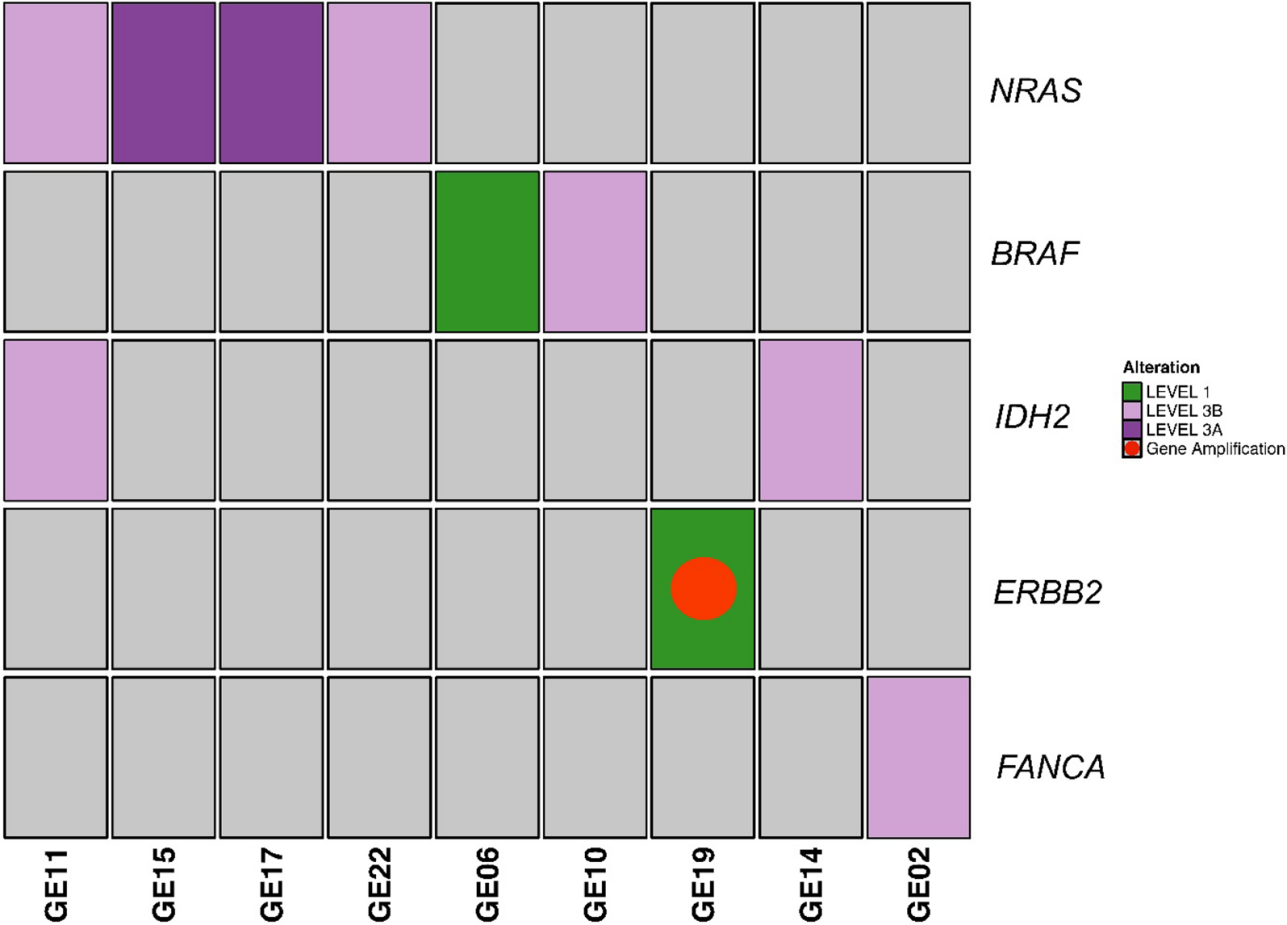
Actionable gene variants in 9 out of 23 circulating cell-free DNA samples analyzed. Actionable somatic variants and gene amplifications (OncoKB level 1–3) in 9/23 (39.1%) circulating cell-free DNA samples are reported. OncoKB level 1, 3B, 3A is indicated in green, lilac, purple, respectively. Gene amplification with OncoKB level 1 is represented with a red circle

cfDNA was analyzed for all patients. Among 410 somatic alterations (VAF ≥ 0.1% and VAF ≥ 0.5% for SNVs/MNVs and InDels, respectively) detected in the 23 cfDNAs (Additional file 10), 25 were classified as OncoKB Levels 1–4. Among them, 9 variants classified as level 1–3 (34.8%) and one of them level 1, were identified in 14 samples (60.9%) (Fig. 2 and Table 2). The cfDNA samples showed no microsatellite instability and median TMB of 8.9 ranging from 1.9 to 59.6 mut/Mb. CNV analysis revealed 4 amplifications (FC ≥ 1.5) in cfDNA (Additional files 11 and 12), but only one classified as OncoKB level 1 (*ERBB2* amplification in GE19 sample) (Fig. 2). Overall, a total of 9 somatic mutations and 1 CNV were classified as OncoKB levels 1 to 3 in 9/16 patients (56.25%) (Fig. 2 and Table 2). Eight of them had not been detected by routine testing (*FANCA* p.W911Dfs*31 in GE02 pancreatic cfDNA, 3 *NRAS* p.Q61K in GE17 and GE15 melanoma and in GE11 cholangiocarcinoma, a *NRAS* p.G13D in GE22 pancreatic cancer, an *IDH2* p.R172S in GE11 cholangiocarcinoma, and an *IDH2* p.R140Q in GE14 melanoma).

### Circulating cell-free DNA (cfDNA) mutational profiles and dynamic changes during treatment in GE01

**Fig. 3:**
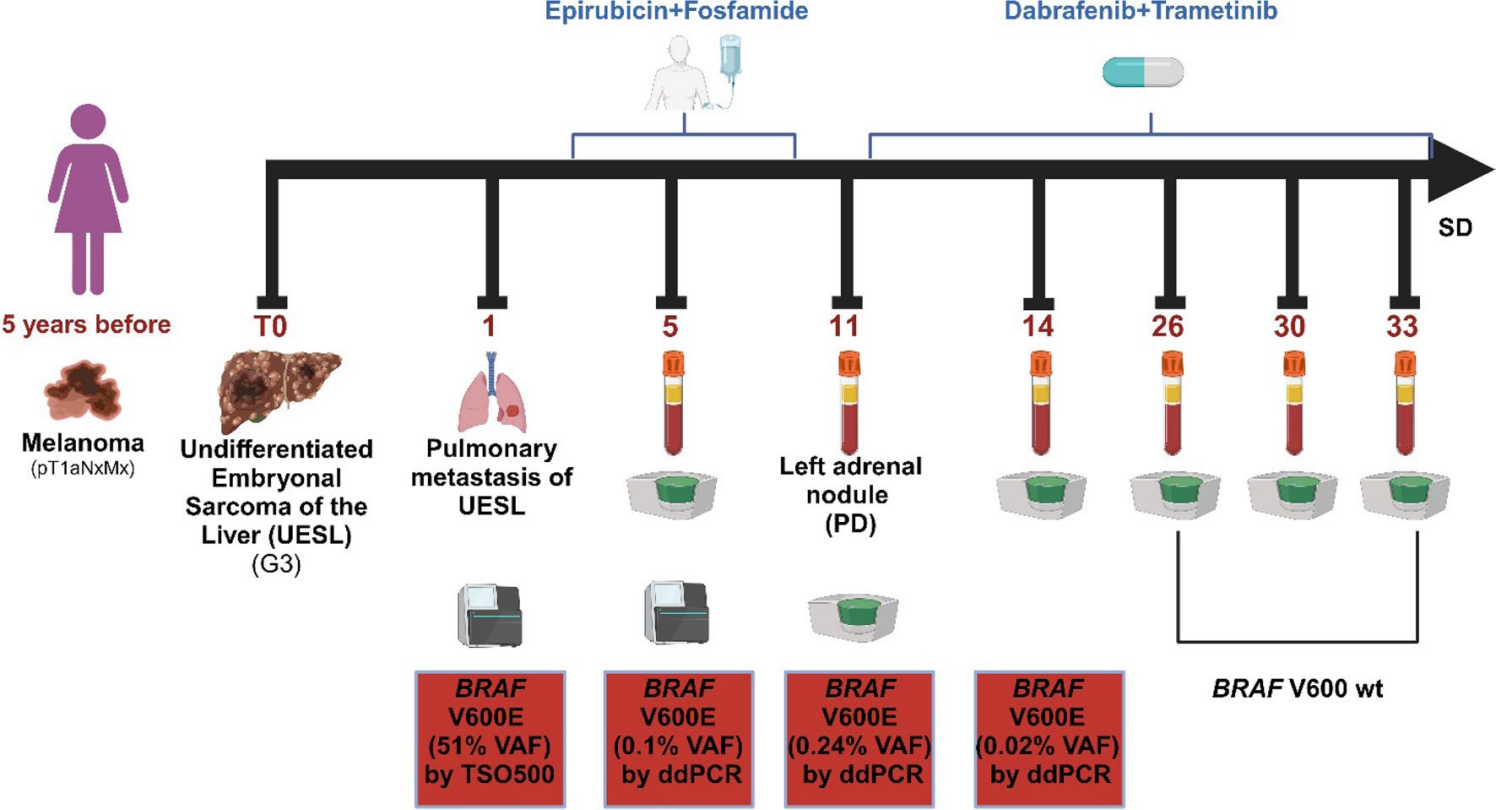
Circulating cell-free DNA mutation profiles and dynamic changes during treatment of GE01 patient. Timeline of the patient’s clinical history and longitudinal tracking of circulating cell-free DNA are shown. Time points on therapy are indicated with blue brackets. The timeline baseline is indicated as T0 and the subsequent times are calculated in months starting from baseline. *UESL* undifferentiated embryonal sarcoma of the liver, *PD* progressive disease, *SD* stable disease, *wt* wild-type, *VAF* variant allele frequency

For the GE01 patient, we were able to assess dynamic changes during treatment by analyzing cfDNA at five consecutive time points. The patient was diagnosed with a superficial spreading melanoma, Breslow thickness of 0.63 mm, Clark level II, with no ulceration, pT1aNxMx, at the 33 years of age. After 5 years, the patient developed an Undifferentiated Embryonal Sarcoma of the Liver (UESL) (G3) and underwent right hepatectomy. A histological review of the liver mass was requested and confirmed the diagnosis of embryonal liver sarcoma. Surprisingly, the TS0500 Tissue analysis revealed the *BRAF* V600E mutation (classified as OncoKB level 3A or Tier IA) with a VAF of 51% on pulmonary metastatic lesion of UESL, which was undetected by the TSO500 cfDNA analysis performed about 5 months later (after pulmonary metastasectomy for the lung metastases), while ddPCR analysis showed one droplet positive on plasma (VAF of 0.1%; 8.1 ng/1 mL). After about 6 months, due to the appearance of a left adrenal nodule at progression, BRAF plus MEK inhibitors treatment (Encorafenib + Binimetinib) was started. Concurrently, ddPCR on cfDNA was repeated on a new plasma sample confirming the presence of *BRAF* V600E with a VAF of 0.24% (2.4 ng/1 mL of starting plasma). After that, cfDNA analysis was carried out by ddPCR at 4 subsequent serial points during treatment, confirming the absence of the mutation in the last 2 points (Fig. 3).

In confirmation of the liquid biopsy analysis, the patient is currently in good general condition (ECOG Scale of Performance Status 0). The computed tomography further supports the stable disease, showing no change in the previously identified thickening of the anterior mediastinum in the thymic area and left adrenal nodule. Additionally, it revealed a reduction in size of some solid nodules in the adipose tissue between the right kidney and ascending colon (3 × 2 mm *vs* 6 × 3 mm). Considering the response to therapy, the patient is continuing with the combination of BRAF and MEK inhibitors treatment.

### Germline pathogenetic/likely pathogenetic variants detection yield

For all recruited patients, gDNA were evaluated with the TruSight™ Hereditary Cancer Panel (Illumina) and raw data were analysed by Emedgene platform (Illumina). Among 8271 germline variants detected in the 23 gDNAs (Additional file 13), the analysis revealed 3237 exonic, 2576 intronic, 22 downstream, 1772 intergenic, 212 ncRNA intronic/esonic, 24 splicing, 428 UT3/UTR5 variants; among these, 6 pathogenetic/likely pathogenetic variants (1 *BRCA1*, 1 *VHL*, 2 *ATM*, 1 *MSH3* and 1 *MUTYH*) were identified (Table 2).

Moreover, germline-focused analysis of tumor-detected variants by TSO500 panels called 2394 and 3634 germline variants in the 16 tumor tissues and in the 23 cfDNAs samples, respectively (Additional files 14 and 15). In the 16 tumor tissues matched-cfDNA, 2529 cfDNA germline variants were found (Additional file 16); among them, 1587 germline variants were in common between the tissue and matched-cfDNA (Additional file 17). All the 6 pathogenetic/likely pathogenetic variants identified by the TruSight™ Hereditary Cancer Panel were found both in circulation and in tissue, when analysed (Table 2). Only 1 out of 6 pathogenetic/likely pathogenetic variants (*VHL* p.N131T) was from a patient with suspected hereditary cancer syndrome (VHL syndrome). The remaining 5 pathogenetic/likely pathogenetic variants (*BRCA1* p.C64R, *ATM* p.S1993Rfs*23 and p.A1299Cfs*3, *MSH3* p.Q29X, and *MUTYH* p.G368D) occurred in patients with apparently sporadic cancer. In addition, the germline-focused tumor analysis revealed a *CHEK1* frameshift pathogenic variant (NM_001114122: p. E320Nfs*36, c.958delG, Exon 10); the *CHEK1* gene is not covered by the TruSight™ Hereditary Cancer Panel design (Additional file 1). This *CHEK1* variant was also validated by Sanger Sequencing on a germline DNA sample. The overall germline variants with an evidence of clinical actionability are reported in Table 3. In particular, 5 germline variants (1 *BRCA1*, 1 *VHL*, 1 *CHEK1,* and 2 *ATM* variants), classified as OncoKB level 3B, were identified; all carriers had not been previously tested, but only the patient with the *VHL* p.Asn131Thr variant met genetic testing criteria.

**Table 3:** Therapeutically actionable somatic/germline variants in study cohort

Patient ID	Gene	Refseq	HGVS	OncoKB -AMP/ASCO/CAP classification	cfDNA/tumor tissue/blood
GE01●	*BRAF^*	NM_004333.4	p.V600E	1/IA	Tumor tissue
GE02	*FANCA^*	NM_000135	p.W911Dfs*31	3B/IIC	cfDNA
GE05	*BRCA1°^*	NM_007294.3	p.C64R	3B/IIC	cfDNA/blood
GE06●	*BRAF*	NM_004333.4	p.V600R	1/IA	Tumor Tissue/cfDNA
GE10●	*BRAF*	NM_004333.4	p.V600K	3B/IIC	cfDNA
GE11	*IDH2^*	NM_002168.4	p.R172S	3B/IIC	cfDNA
	*NRAS^*	NM_002524.4	p.Q61K	3B/IIC	cfDNA
GE12●	*VHL°^*	NM_000551.3	p.N131T	3B/IIC	Tumor tissue/cfDNA/blood
GE14●	*IDH2^*	NM_002168.4	p.R140Q	3B/IIC	cfDNA
	*BRAF*	NM_004333.4	p.V600E	1/IA	Tissue
	*CHEK1°^*	NM_001330427	p.E336Nfs*36	3B/IIC	Tumor tissue/cfDNA/blood
GE15	*NRAS^*	NM_002524.4	p.Q61K	3A/IB	Tumor tissue/cfDNA
	*ATM°^*	NM_000051.4	p.S1993Rfs*23	3B/IIC	Tumor tissue/cfDNA/blood
GE17	*NRAS^*	NM_002524.4	p.Q61K	3A/IIB	cfDNA
GE19●	*ERBB2*	NM_000051.4	Amplification	1/IA	Tumor tissue/cfDNA
GE21	*ATM°^*	NM_000051.4	p.A1299Cfs*3	3B/IIC	Tumor tissue/cfDNA/blood
GE22●	*NRAS^*	NM_002524.4	p.G13D	3B/IIC	cfDNA

Different type of genomic actionable alterations according to OncoKB or AMP/ASCO/CAP classification (1–3 or IA-IIC levels) are shown. Patients ID with tumor tissue collected within 4 months from plasma collection are indicated with a black circle. Germline and somatic variants not previously analysed by routine testing are reported with ° and ^, respectively

*cfDNA* circulating cell-free DNA, *HGVS* Human Genome Variation Society nomenclature

### Therapeutic actionability yield

In order to recover as many clinically actionable variants as possible, mutational calls were fed into the Illumina Connected Analytics (ICA) and OncoKB version 4.0 pipeline for AMP/ASCO/CAP and for OncoKB classifications, respectively [15–17]. The integrated tissue and cfDNA analysis led to the identification of strong or potential clinical significance variants/CNVs in 43.5% of patients (10/23 patients) (Table 3); in 8 of these 10 patients (34.8% of the patients cohort), 8 somatic variants (*FANCA* p.W911Dfs*31 in GE02, *NRAS* p.Q61K in GE11, GE17 and GE15, *NRAS* p.G13D in GE22, *IDH2* p.R140Q in GE14, *IDH2* p.R172S in GE11, *BRAF* V600E in GE01) had not previously been detected by routine testing. Germline analysis showed pathogenic/likely pathogenic variants in 7/23 (30.4%) patients (Table 2), 5 of which were classified as OncoKB levels 3B in 5/23 (21.7% patients) (Table 3). The overall actionability yield reached, considering the combination of somatic and germline variants/CNVs, allowed to identify at least one somatic or germline actionable variant in 56.5% of patients (13/23). However, considering the combination of somatic and germline variants/CNVs, not previously identified by somatic and germline routine testing, the overall therapeutically detection yield was 43.5% (10/23 patients).

## Discussion

Cancer molecular profiling using NGS comprehensive panels has been implemented in clinical practice, but still presents challenges and unresolved issues. To this regard, multiple international medical oncology societies recently proposed their recommendations for the use of NGS [2, 23, 24]. Indeed, in the real-world clinical practice, NGS panels are being increasingly requested by oncologists, although the utility of upfront full molecular profiling in all cancer patients is still a matter of dispute [25]. Selection criteria across different MTB are not standardized and specific guidelines are not available, although real-world MTB experiences as well as position papers from scientific societies addressing this issue are increasingly being published [26–29].

Another point of debate is whether ctDNA sequencing with large panels could represent a viable alternative to conventional tissue biopsy. Indeed, adequately powered studies are needed to understand whether tumor tissue DNA (tDNA)-only testing could be replaced by ctDNA-first, ctDNA-only, or fully parallel tDNA/ctDNA testing schemes [28, 30]. Moreover, germline testing following somatic sequencing findings, as part of clinical assessment, is also becoming increasingly relevant. The clinical actionability of germline findings may impact patient care in terms of tailored medical/surgical treatment, as well as genetic counselling for cancer risk assessment for both patients and their relatives [31–33].

In this study, we sought to determine the actual detection yield of actionable variants in our real-world cohort of patients, who may benefit most from tailored therapy and/or prevention. We also addressed the capacity of our integrated genomic profiling to uncover hereditary predisposition, as well as the appropriateness of liquid biopsy instead of, or in addition to, tumor tissue analysis.

First of all, we addressed the identification of the lowest VAF cut-off for both bulk tumor and ctDNA analysis using the TruSight Oncology panels, to obtain confident variants exploiting the peculiarity of our cohort. Similarly to recent studies [21, 22, 34, 35], we opted to consider all variants with a VAF ≥ 5% for the tumor tissue DNA, ≥ 0.1% for the cfDNA SNVs/MNVs, and ≥ 0.5% for cfDNA InDels, based on JI calculation (JI median = 0.89 ± 0.03) and InDels manual revision. With Jaccard Index, we also provided a measurement of concordance between the two tests. Our experience showed that almost all the variants were shared when considering the 5%-0.5% cut-offs (solid-cfDNA), with the variability in the concordance that was clearly patient-dependent, as demonstrated by GE6, characterized by a low concordance (JI = 0.82) and, conversely, by the large overlapping in variant calling for GE12 (JI = 0.93), GE19 (JI = 0.93) and GE22 (JI = 0.92).

In general, once a variant has been identified, interpretation of pathogenicity and actionability is of key importance, and still represents a challenge in precision oncology. This critical aspect is negatively affected by the increasing scale and complexity of molecular data generated by the complete sequencing of cancer samples which requires advanced interpretative platforms. In our study, for the interpretation of clinically actionable somatic variants, each variant was classified by OncoKB criteria and subsequently re-analysed with Illumina Connected Insights (ICI) software which was able to correctly call the variants in the corresponding AMP/ASCO/CAP Tier.

In our study, gene variants were classified and attributed to the corresponding OncoKB level or AMP/ASCO/CAP Tier using two independent software callers obtaining overlapping results, in order to strengthen the reliability of our data interpretation.

Typically, extended genomic profiling is performed on either tumor tissue or cfDNA, and possible secondary germline findings need confirmation by germline testing. Here, we assessed germline status upfront for all patients, confirming germline variant predictions from somatic testing at tumor and/or liquid biopsy level.

Overall, our integrated cfDNA and tumor analysis revealed clinically actionable variants/CNVs in 43.5% of patients (10/23 patients), increasing up to 56.5% when germline analysis results (13/23 patients had at least one somatic or germline actionable variant) were included. A subset of the clinically actionable variants had been previously detected by somatic and germline routine testing, so the actual overall detection yield of actionable variants obtained considering the combination of novel somatic and germline variants/CNVs was 30.1% (9/23 patients). Considering only the combined analysis of matched-tumor sampling within 4 months and cfDNA, 5 clinically actionable variants would have not been identified, suggesting the clinical relevance of performing parallel tDNA/ctDNA testing, when possible [28].

In particular, only 2 of these, both *BRAF* p.V600E, were only detected in the tumor: one in a melanoma patient (GE14) who had a brain metastasis and the other one in a sarcoma patient (GE1) undergoing metastasectomy for the lung metastases. The lack of concordance between cfDNA and DNA extracted from tissue biopsy is not uncommon [36, 37]. Finally, the analysis of cfDNA in patients with paired tumor tissue available, collected > 4 months prior to plasma, showed the disappearance of actionable variants, likely due to therapy response and clonal evolution, as already widely reported [38].

**Fig. 4:**
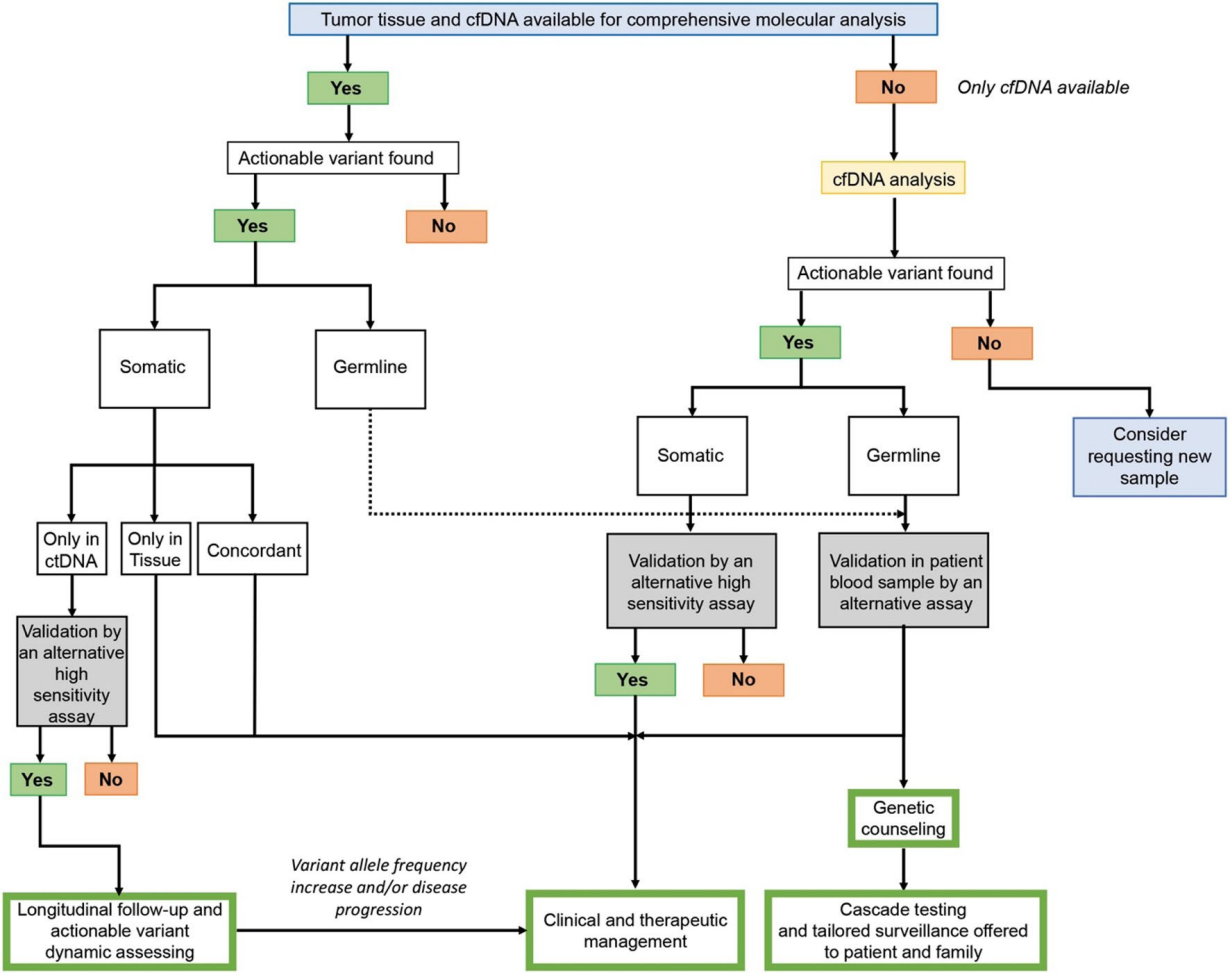
Workflow to improve the detection yield of actionable variants and their implications for cancer patient’s outcome and clinical management. The flow chart suggests the use of an integrated cfDNA, tumor and germline analysis by next-generation sequencing comprehensive panels in order to increase the yield of actionable variants. The suggested workflow is based on our results obtained in 23 patients with diverse and rare tumor types. For this reason, consider confirming these findings on larger cohorts and prospective clinical trials to support this approach. Our recommendation is to perform the concurrent tumor tissue and cfDNA molecular profiling, when possible. Identification of germline actionable variants is possible through a germline-focused analysis of tumor/cfDNA using a robust and sensitive bioinformatic analysis, opening to genetic counselling and secondary germline testing protocols, as well as cascade testing in family members. The figure also addresses the interpretation of discordant data between tumor tissue biopsy versus liquid biopsy and their clinical implications. While actionable variants found only in tumor DNA and both in tumor DNA and cfDNA are candidate for clinical and therapeutically management, we suggest that actionable variants found only in ctDNA need further confirmation with orthogonal sensitive methods and, if confirmed, longitudinal monitoring is indicated, prior to consideration for therapy treatment and implications on patient outcomes

Although variant detection by both testing methods was largely concordant, concordance rates varied considerably by cancer subtype. Spatial heterogeneity is likely a dominant biological factor influencing the detection of ctDNA-unique and solid-tissue unique variants, either through intratumor heterogeneity, or intertumor heterogeneity across metastatic lesions [39]. Discordance between mutations identified in tumor tissue and ctDNA has been already described and can be attributed to tumor heterogeneity or evolution, sampling bias, time lapses between samples acquisition, and differences in sensitivity of the NGS assays employed [40, 41]. Based on our study results, we suggest an algorithm to enhance identification, by extended combined genomic profiling, of actionable variants for both therapeutic and preventive approaches. This algorithm also addresses challenges and solutions for integrating these findings into clinical workflows (Fig. 4).

Among all the cases, GE01 was emblematic of the utility of our extended genomic profiling for both therapeutic strategies changes and for monitoring response to therapy through cfDNA analysis. In this patient an unexpected *BRAF* p.V600E mutation (OncoKB level 1) was found in a lung metastasis from UESL and confirmed on matched-cfDNA by ddPCR but not by TS0500 500 ctDNA panel. This discrepancy seems to be related to the fact that the patient had a low disease burden after surgical removal of the metastasis, confirmed by low levels of cfDNA, and that the ddPCR technique is more sensitive than TS0500 ctDNA panel analysis, albeit in a stochastic manner. Indeed, the ddPCR analysis identified only one *BRAF* p.V600E-positive droplet. Longitudinal analysis of ctDNA, at 4 subsequently time points, by ddPCR showed a progressive disappearance of this mutation following the treatment with BRAF + MEK inhibitors, in line with the patient showing stable disease, thus confirming the clinical utility of this targeted approach in sarcoma treatment [42, 43]. *BRAF* V600 mutations are detected in ~ 7–15% of all cancers, although with different prevalence in different cancer types, including hairy cell leukemia (79–100%), melanoma (40–70%), papillary thyroid cancer (45%), ovarian cancer (35%), colorectal cancer (11%), cholangiocarcinoma (5–7%), multiple myeloma (4%) and non-small cell lung cancer (1–3%), but also in rare and very rare cancers, such as sarcomas, among others [44]. To the best of our knowledge, this is the first report of a BRAF-mutated UESL successfully treated with BRAF and MEK inhibitors, suggesting the possibility of using the *BRAF* p.V600E mutation as a therapeutic target in patients with UESL and confirming the clinical relevance of comprehensive genomic profiling.

In addition, 5 germline pathogenic variants (American College of Medical Genetics and Genomics-ACMG class 5) were also classified as level 3B according to OncoKB (Table 3). Of those, only the *VHL* variant occurred in a patient, previously untested, but fulfilling the criteria for genetic testing. Interestingly, this variant was therapeutically actionable (OncoKB level 3B), but target drugs are not universally available (only Food and Drug Administration (FDA)-approved) [45, 46]. All the others pathogenic variants were from patients not meeting criteria for germline testing for the specific genes, but their identification uncovered an underlying hereditary cancer predisposition. Germline pathogenic variants in *ATM*, such as the one identified in GE21, are known to increase the risk of pancreatic cancer, but germline testing for this gene is not routinely performed [47, 48]. This patient had no family history of pancreatic cancer (apparently sporadic cancer) at the time of study inclusion. The second *ATM* germline variant carrier (GE15), was a patient affected by cutaneous melanoma. We previously proposed *ATM* (including this variant), as a candidate melanoma predisposition gene [36, 49]. Interestingly, there are some data supporting that *ATM* variant carriers diagnosed with pancreatic ductal adenocarcinoma could have longer survival [48, 50], and both *ATM* variants here identified were found in two long responding patients diagnosed with melanoma and pancreatic cancer, respectively.

Whether *ATM* variants could be targets of a specific therapeutic approach in these conditions is still unclear [50, 51]. The *BRCA1* and *CHEK1* variants were identified in a rare breast neuroendocrine tumor (GE05) and in a melanoma case (GE14), respectively. Enrolment in clinical trials could be an option for the treatment of these patients (NCT04633902; NCT02873975; NCT05787587; NCT06022029).

With regard to the *BRCA1* germline variant found in a patient affected by a rare breast neuroendocrine tumor, genetic testing revealed a shared *BRCA1*-related cancer predisposition in this family: genetic counselling was followed by cascade testing, revealing a family member affected by breast cancer carrying the same pathogenic variant. This case is emblematic of clinical implications of identifying previously undetected germline variants, particularly in terms of cancer risk assessment and genetic counselling.

Our approach uncovered hereditary predisposition independently on genetic testing criteria, in line with recent research data [52–56]. Indeed, the identification of previously undetected germline pathogenic variants opens to genetic counselling and cascade testing to family members. A tailored cancer risk assessment, and inclusion in prevention protocols and tailored surveillance, should be done in the context of certified centers for genetic testing and in the framework of multidisciplinary management (i.e. Molecular Tumour Boards).

Overall, our tumor/cfDNA profiling showed reliability in correctly uncovering germline susceptibility as demonstrated by overlapping results of parallel germline and somatic sequencing.

In the era of precision oncology, germline and tumor/cfDNA profiling is increasingly being implemented in clinical practice. This approach has been successful in the molecular characterization of various tumor types, leading to major improvements concerning prognostic assessment, therapeutic actionability and/or preventive protocols. However, the future of precision medicine will likely integrate comprehensive multi-omic tumor characterization, dynamic monitoring of liquid biopsy samples, automated variant annotations with artificial intelligence and machine learning approaches and experts’ clinical input [57–59].

Our study has several limitations. First, the retrospective nature of the study and the diverse tumor types introduce considerable variability, necessitating larger cohorts/studies and prospective clinical trials to validate the mutation detection rates and their clinical relevance across various and rare cancers. Second, the absence of a control group undergoing standard treatment protocols, requires a cautious approach when interpreting the study's findings and their applicability. Finally, we could only partially explore the implications of our findings for clinical practice in these patients, since, due to the small number of patients and their clinical conditions, not all the actionable findings were translated in clinical practice changes.

## Conclusions

Although our real-world study was conducted on a small and heterogeneous cohort, we point out evidence that our integrated cfDNA, tumor and germline analysis allowed us to discover actionable variants in more than half of our cohort.

These findings pave the way to the possible implementation of comprehensive genomic profiling, always after discussion within a multidisciplinary MTB context in order to identify not only somatic, but also germline variants for tailored management of cancer patients.

In conclusion, this study confirmed the clinical relevance of extended tumor/cfDNA testing, not only for broadening therapeutic options, but also for monitoring treatment efficacy and revealing genetic predisposition in patients, underscoring the potential role of comprehensive genomic profiling in shaping the future of cancer therapy and prevention.

Studies specifically addressing whether this approach, performed within MTBs, could lead to improve treatment efficacy, mitigate adverse effects, or enhance the quality of life for cancer patients are needed. Indeed, MTB- led case–control clinical trials with adequate power are limited [60]. Indeed, preliminary data show that MTB-managed patients receiving genomic profiled recommended therapy showed improved Progression-free survival/clinical outcomes [60–63]. Future research should concentrate on prospective trials and standardization of approaches and reporting of clinical outcomes.

### Supplementary Information

**Additional file 1. **Genes and their alterations types covered by next-generation sequencing panels used in the study. The genes and their alterations covered by TruSight Oncology 500 tumor/ctDNA panel and TruSight Hereditary Cancer Panel are shown. The private genes and the common genes included in each next-generation sequencing panels used are also indicated.

**Additional file 2. **Evaluation of the best cut-off for using the Jaccard Index (JI) parameter. **a** Distribution plots reporting the JI (Y-axis) post-hoc pairwise comparisons between each VAF intersection. X-axis labels showed the different VAF intersections, composed by a conserved code: tXX_ctYY, where t stands for “TSO500 solid tumor” and ct for “TSO500 ctDNA”, followed by the respective VAF cut-off. Each point represented the JI for a single patient, painted with a specific color. At the top of the boxplots, we only reported the pairwise correlations (p < 0.05). **b** Line plots reporting the patient-specific, JI (Y-axis) trend at the different VAF intersections. X-labels are coded as in the figure above.

**Additional file 3. **Tukey comparison plot between each intersection of TruSight™ Oncology 500 (TSO500) solid Variant Allele Frequency (VAF) and TruSight™ Oncology 500 (TSO500) ctDNA Variant Allele Frequency (VAF). Tukey comparison plot between each intersection of TSO500 solid tumor VAF and TSO500 ctDNA VAF is shown. The first column reported the comparison (with the same code ID reported in additional file 2 Figure S1), and the adjusted p-value with fdr in the last column. ns: not significant; t: tumor; ctDNA: circulating-free tumor DNA.

**Additional file 4. **Tumor Mutational Burden (TMB) and Microsatellite Instability (MSI) for each tumor tissue and cfDNA analyzed in the study. Tumor Mutational Burden (TMB) and Microsatellite Instability (MSI) for each tumor tissue and circulating free-DNA belonging to each patient including in the study are shown. Samples ID with tumor tissue with TMB ≥ 10 mutations/Mb are indicated with asterisks. n.d.: not done; TMB: Tumor Mutational Burden; cfDNA: circulating- free DNA; MSI: MicroSatellite Instability.

**Additional file 5. **The somatic alterations (variants allele frequency ≥ 5%) found in the 16 tumor tissues analyzed in the study. The somatic alterations (coding/nonsynonymous) detected with a variant allele frequency ≥ 5% in the 16 tumor samples analyzed by the TruSight™ Oncology 500 panel are shown. For each patient, the somatic alterations are annotated.

**Additional file 6. **The somatic alterations, classified as OncoKB level 1 to 4, in the 16 tumor tissues analyzed in the study. The 11 somatic alterations (coding/nonsynonymous variants with a variant allele frequency ≥ 5%), classified as OncoKB level 1 to 4, identified in the 16 tumor tumous analyzed by the TruSight™ Oncology 500 panel are shown. For each patient, the somatic alterations, classified as OncoKB level 1 to 4, were annotated. In bold are indicated the 6 somatic alterations classified with OncoKB level 1 to 3.

**Additional file 7. **Copy number variations fold-changes for each gene analyzed by TruSight™ Oncology 500 panel in the 16 tumor tissues. Copy number variations fold-change for each tumor tissue belonging to each patient including in the study are shown. In bold are indicated the fold-change ≥ 2.

**Additional file 8. **Heatmap of the Copy Number Variants (CNVs) obtained from the TSO500 solid tumor sequencing. Fold-Change (FC), with a color scale from blue (loss) to red (gain), is reported. Column dendrogram groups co-occurrent CNVs.

**Additional file 9. **Amplifications with a fold-change ≥ 2 in the 16 tumor tissues analyzed in the study. The 4 potential amplifications with a fold-change ≥ 2 in the 16 tumor tissues analyzed by TruSight™ Oncology 500 panel are shown. In bold is indicated the tumor tissue classified with OncoKB level 1 or Tier IA.

**Additional file 10. **The somatic alterations (VAF ≥ 0.1% and VAF ≥ 0.5% for SNVs/MNVs and InDels, respectively) found in the 23 circulating-free DNAs analyzed in the study. The somatic alterations (coding/nonsynonymous), detected with a VAF ≥ 0.1% for SNVs/MNVs and VAF ≥ 0.5% for InDels, in the 16 tumor tumors analyzed by the TruSight™ Oncology 500 ctDNA panel are shown. For each patient, the somatic alterations were annotated.

**Additional file 11. **Copy number variations fold-changes for each gene analyzed by TruSight™ Oncology 500 ctDNA panel in the 23 circulating-free DNAs. Copy number variations fold-change for each circulating-free DNA belonging to each patient including in the study is shown. In bold are indicated the fold-changes ≥ 1.5.

**Additional file 12. **Heatmap of the Copy Number Variants (CNVs) obtained from the TSO500 ctDNA sequencing. Fold-Change (FC), with a color scale from blue (loss) to red (gain), is reported. Column dendrogram groups co-occurrent CNVs.

**Additional file 13. **Germline variants detected in the 23 genomic DNAs by the TruSight^TM^ Hereditary Cancer Panel. The 8271 germline variants, detected in the 23 genomic DNAs by TruSight^TM^ Hereditary Cancer Panel, are shown. For each patient, the germline variants are annotated.

**Additional file 14. **Germline variants found in the 16 tumor tissues by the TruSight^TM^ Oncology 500 panel. For each patient, the germline variants are annotated.

**Additional file 15. **Germline variants found in the 23 circulating- free DNAs by the TruSight^TM^ Oncology 500 ctDNA panel. For each patient, the germline variants are annotated.

**Additional file 16. **Germline variants found in the 16 circulating-free DNAs matched-tissue tumor samples by the TruSight^TM^ Oncology 500 ctDNA panel. For each patient, the germline variants are annotated.

**Additional file 17. **Germline variants found in common between the 16 tumor tissues and matchedcirculating-free DNAs by the TruSight^TM^ Oncology 500 panels.

## Data Availability

The datasets generated and/or analysed during the current study are not publicly available but are available from the corresponding author on reasonable request.

## Abbreviations

ACMG: American College of Medical Genetics and Genomics
cfDNA: Circulating cell-free DNA
ctDNA: Circulating tumor DNA
CPs: Comprehensive panels
CN: Copy-number
CNVs: Copy number variations
ddPCR: Droplet digital PCR
FC: Fold-change
FDA: Food and Drug Administration
FFPE: Formalin-fixed paraffin-embedded
gDNA: Genomic DNA
ICA: Illumina connected analytics
InDels: Insertions/deletions
JI: Jaccard index
MSI: MicroSatellite Instability
MTBs: Molecular Tumour Boards
MNVs: Multi-nucleotide variants
NGS: Next-generation sequencing
SNPs: Single-nucleotide polymorphisms
SNVs: Single nucleotide variants
TSO: TruSight oncology
TMB: Tumor mutational burden
UESL: Undifferentiated embryonal sarcoma of the liver
VAF: Variant allele frequency
