# Supplementary figures and images for: Combining germline, tissue and liquid biopsy analysis by comprehensive genomic profiling to improve the yield of actionable variants in a real-world cancer cohort
Source: J Transl Med. 2024 May 15;22:462. doi: 10.1186/s12967-024-05227-2 (PMC11097509; doi:10.1186/s12967-024-05227-2)

$p < 0.05$

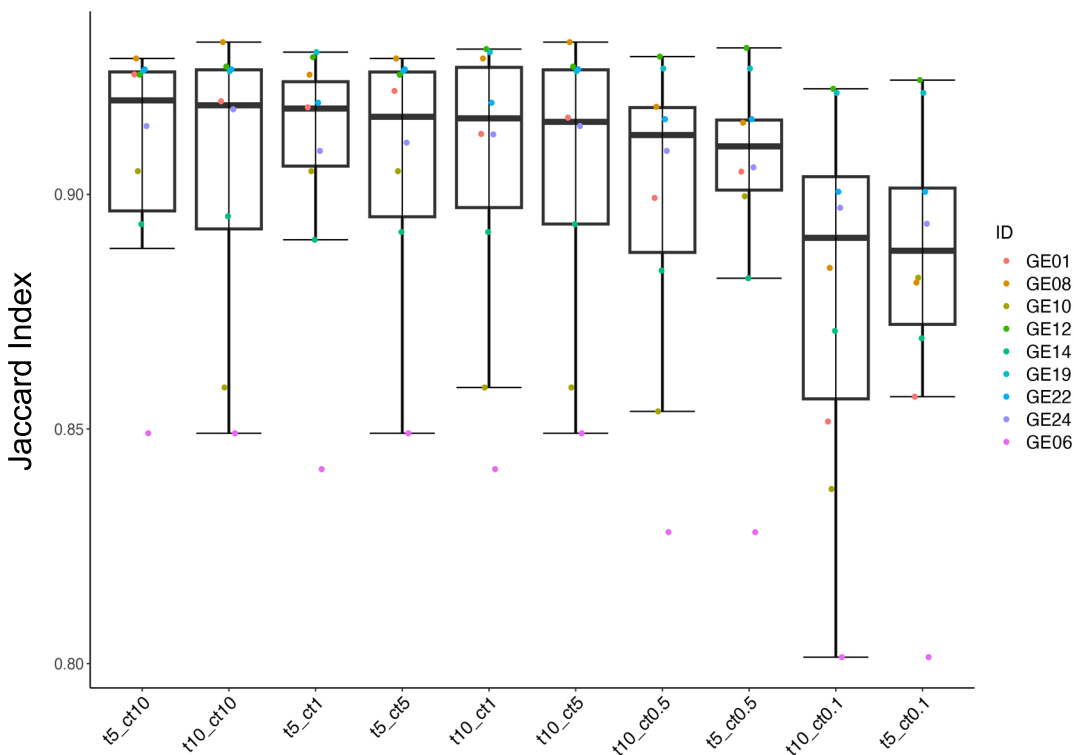

**b**

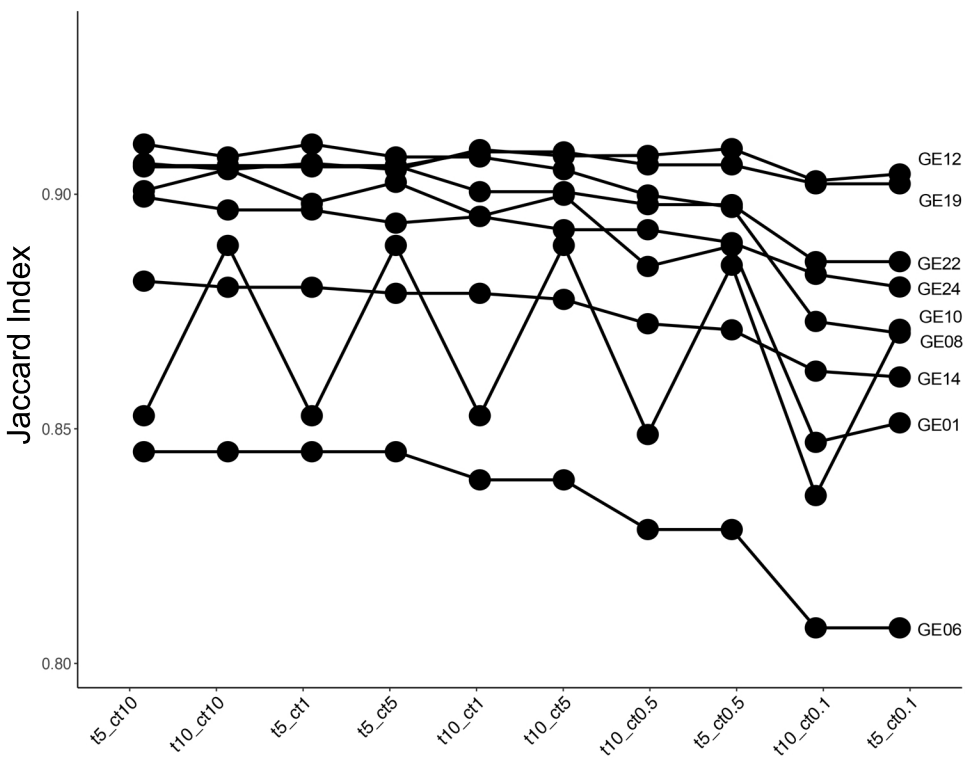

Supplement: Supplementary file 2 — Additional file 2. Evaluation of the best cut-off for using the Jaccard Index (JI) parameter. a Distribution plots reporting the JI (Y-axis) post-hoc pairwise comparisons between each VAF intersection. X-axis labels showed the different VAF intersections, composed by a conserved code: tXX_ctYY, where t stands for “TSO500 solid tumor” and ct for “TSO500 ctDNA”, followed by the respective VAF cut-off. Each point represented the JI for a single patient, painted with a specific color. At the top of the boxplots, we only reported the pairwise correlations (p < 0.05). b Line plots reporting the patient-specific, JI (Y-axis) trend at the different VAF intersections. X-labels are coded as in the figure above. [file 12967_2024_5227_MOESM2_ESM.pdf]

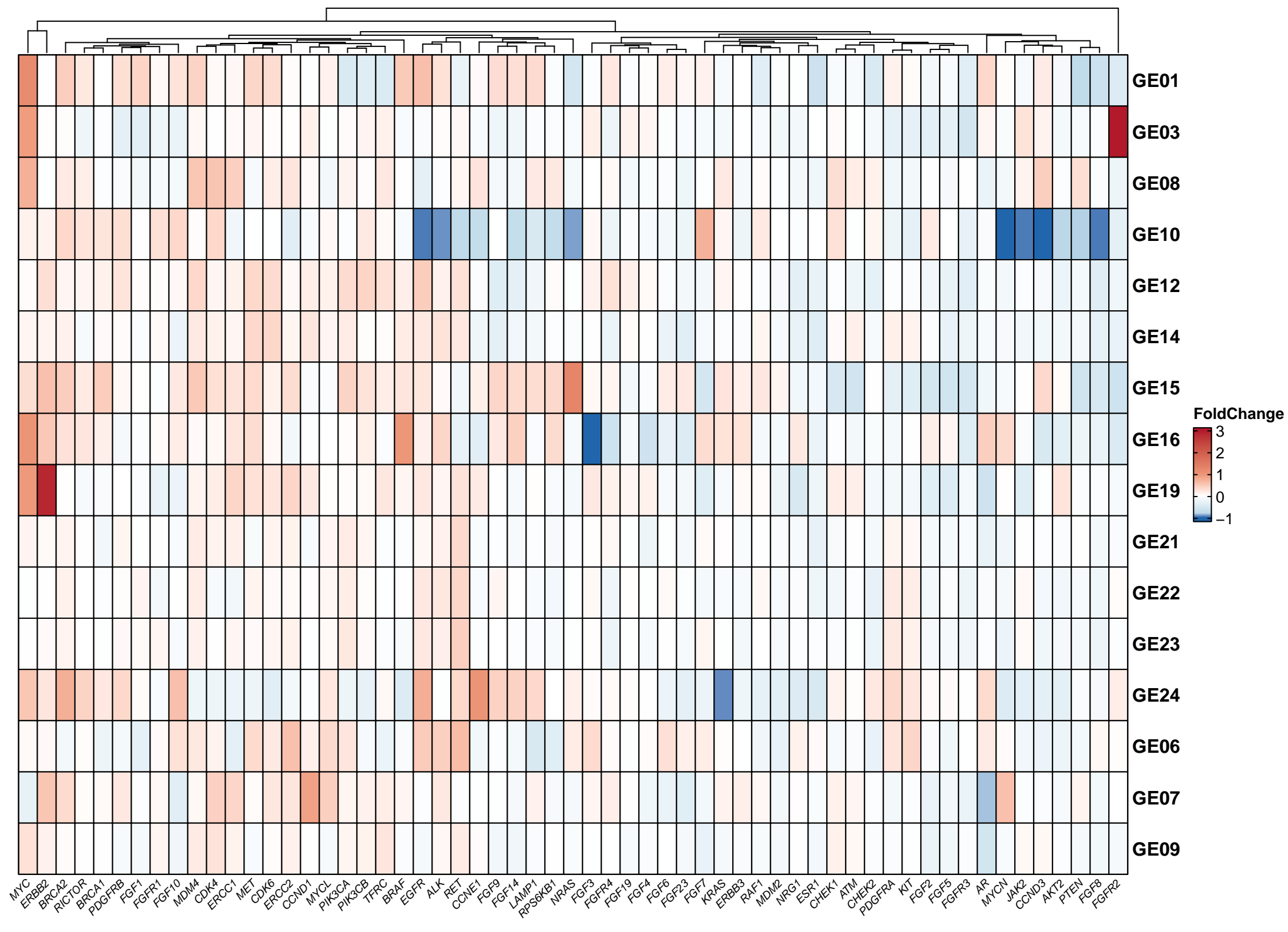

Supplement: Supplementary file 8 — Additional file 8. Heatmap of the Copy Number Variants (CNVs) obtained from the TSO500 solid tumor sequencing. Fold-Change (FC), with a color scale from blue (loss) to red (gain), is reported. Column dendrogram groups co-occurrent CNVs. [file 12967_2024_5227_MOESM8_ESM.pdf]

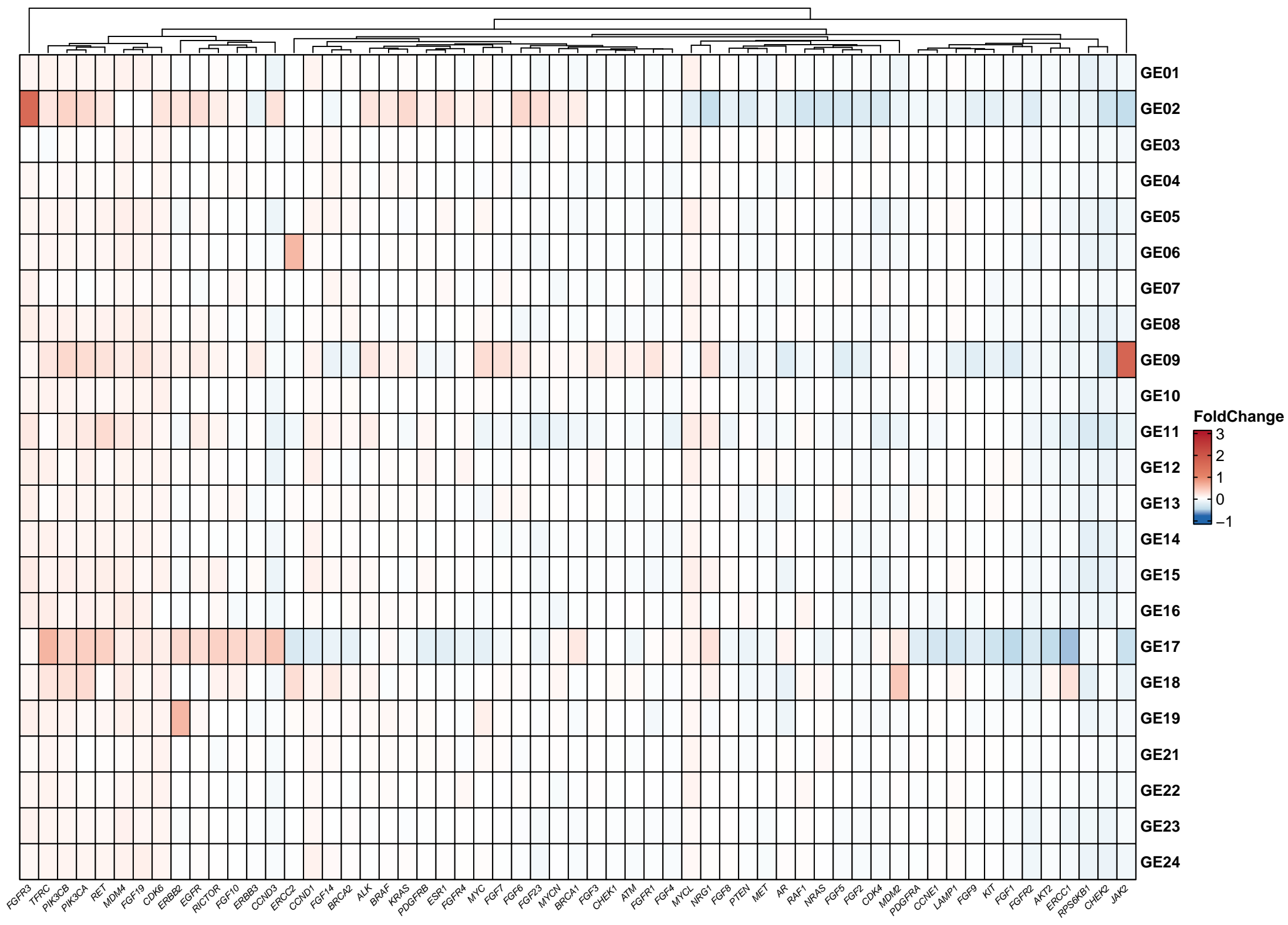

Supplement: Supplementary file 12 — Additional file 12. Heatmap of the Copy Number Variants (CNVs) obtained from the TSO500 ctDNA sequencing. Fold-Change (FC), with a color scale from blue (loss) to red (gain), is reported. Column dendrogram groups co-occurrent CNVs. [file 12967_2024_5227_MOESM12_ESM.pdf]
